# Relationship between disorders of the intestinal microbiota and heart failure in infants with congenital heart disease

**DOI:** 10.3389/fcimb.2023.1152349

**Published:** 2023-03-10

**Authors:** Qi-Liang Zhang, Xiu-Hua Chen, Si-Jia Zhou, Yu-Qing Lei, Jiang-Shan Huang, Qiang Chen, Hua Cao

**Affiliations:** ^1^ College of Clinical Medicine for Obstetrics & Gynecology and Pediatrics, Fujian Medical University, Fuzhou, China; ^2^ Fujian Maternity and Child Health Hospital College of Clinical Medicine for Obstetrics & Gynecology and Pediatrics, Fujian Medical University, Fuzhou, China; ^3^ Fujian Children’s Hospital (Fujian Branch of Shanghai Children’s Medical Center), College of Clinical Medicine for Obstetrics & Gynecology and Pediatrics, Fujian Medical University, Fuzhou, China; ^4^ NHC Key Laboratory of Technical Evaluation of Fertility Regulation for Non-human Primate, Fuzhou, China

**Keywords:** heart failure, intestinal microbiota, congenital heart disease, disorders of microbiota, infants

## Abstract

**Purpose:**

There is a close relationship between the intestinal microbiota and heart failure, but no study has assessed this relationship in infants with congenital heart disease. This study aimed to explore the relationship between heart failure and intestinal microbiota in infants with congenital heart disease.

**Methods:**

Twenty-eight infants with congenital heart disease with heart failure admitted to a provincial children’s hospital from September 2021 to December 2021 were enrolled in this study. A total of 22 infants without heart disease and matched for age, sex, and weight were selected as controls. Faecal samples were collected from every participant and subjected to 16S rDNA gene sequencing.

**Results:**

The composition of the intestinal microbiota was significantly disordered in infants with heart failure caused by congenital heart disease compared with that in infants without heart disease. At the phylum level, the most abundant bacteria in the heart failure group were Firmicutes, Actinobacteria, Proteobacteria, and Bacteroidetes, and the most abundant bacteria in the control group were Firmicutes, Proteobacteria, Actinobacteria, and Bacteroidetes. At the genus level, the most abundant bacteria in the heart failure group were *Enterococcus*, *Bifidobacterium*, *Subdoligranulum*, *Shigella*, and *Streptococcus*, and the most abundant bacteria in the control group were *Bifidobacterium*, *Blautia*, *Bacteroides*, *Streptococcus*, and *Ruminococcus*. The alpha and beta diversities of the gut bacterial community in the heart failure group were significantly lower than those in the control group (p<0.05). Compared with the control group, retinol metabolism was significantly downregulated in the heart failure group.

**Conclusion:**

Heart failure in infants with congenital heart disease caused intestinal microbiota disorder, which was characterised by an increase in pathogenic bacteria, a decrease in beneficial bacteria, and decreases in diversity and richness. The significant downregulation of retinol metabolism in the intestinal microbiota of infants with heart failure may be related to the progression of heart failure, and further study of the underlying mechanism is needed.

## Introduction

Heart failure is the terminal stage of all types of cardiovascular disease and is a common cause of death (Mosterd and Hoes, 2007; Targher et al., 2017). The pathophysiological mechanisms of heart failure are complex and include haemodynamic abnormalities, neuroendocrine system activation, cardiac remodelling, and inflammatory responses (Mudd and Kass, 2008). To reduce the disease and economic burden associated with heart failure, it is important to elucidate the mechanisms of heart failure development and explore new potential therapeutic targets (Zhang et al., 2021). Accumulating evidence indicates that there is a close relationship between the intestinal microbiota and heart failure. Heart failure can cause a disturbance in the intestinal microbiota, and the intestinal microbiota has potential significance in mediating or regulating the pathophysiology of heart failure (Sandek et al., 2012; Brown and Hazen, 2015; Miele et al., 2015; Marques et al., 2017; Tang et al., 2017; Tang et al., 2019).

Congenital heart disease is a deformity caused by disorders of foetal heart and large blood vessel development (Hoffman and Kaplan, 2002). It is one of the most common congenital malformations and the most common cause of heart failure in children (Hinton and Ware, 2017). Left-to-right shunt congenital heart disease, including patent ductus arteriosus, ventricular septal defect, atrial septal defect, and endocardial pad defect, accounts for approximately 50%–70% of congenital heart disease. Patients with severe disease often develop heart failure in infancy due to a large left-to-right shunt and are seriously ill, which places a heavy burden on families and society. Studies of the relationship between heart failure and the intestinal microbiota have mainly focused on heart disease in adults, and few studies have explored this relationship in infants with congenital heart disease (Ellis et al., 2013). Therefore, we conducted a cohort study of infants with heart failure caused by left-to-right shunt congenital heart disease to explore the relationship between heart failure and the intestinal microbiota. We hypothesised that heart failure in infants with congenital heart disease can cause intestinal microbiota disorder and that this disorder can aggravate the progression of heart failure.

## Methods

### Research design and study cohort

The present study was approved by the ethics committee of our hospital and adhered to the tenets of the Declaration of Helsinki. Additionally, all parents of the patients signed the consent form before participating in the study.

This cohort study aimed to explore the relationship between heart failure and the intestinal microbiota in infants with congenital heart disease. A total of 28 infants with heart failure caused by left-to-right shunt congenital heart disease who were admitted to the cardiac surgery department of a provincial children’s hospital in southeast China from September 2021 to December 2021 were enrolled in this study. A total of twenty-two infants without heart disease and matched for age, sex, and weight were selected as controls.

Inclusion criteria were infants with heart failure caused by left-to-right shunt congenital heart disease. Exclusion criteria the following: (1) other major organ diseases, such as digestive tract malformation, liver failure, or kidney failure; (2) digestive tract diseases, such as diarrhoea, constipation, or jaundice; (3) infection or receiving antibiotics; and (4) parental refusal to participate in the study.

### Faecal sample collection

Faecal samples (1 ml) were collected from each patient, immediately frozen in liquid nitrogen, and stored at −80°C.

### 16S rDNA sequencing

Total genomic DNA samples were extracted using the OMEGA Soil DNA Kit (M5635-02) (Omega Bio-Tek, Norcross, GA, USA). DNA extracted from the sample was used as a template. PCR amplification of the bacterial 16S rRNA genes V3–V4 region was performed using the forward primer 338F (5′-ACTCCTACGGGAGGCAGCA-3′) and the reverse primer 806R (5′-GGACTACHVGGGTWTCTAAT-3′ ). The amplified product was purified and recycled by using clean beads. The purified and recycled products were subjected to fluorescence quantitation with a Quant-iT PicoGreen dsDNA Assay Kit in a microplate reader (BioTek, FLx800). According to the fluorescence quantitation results, each sample was mixed in proportion according to the sequencing volume requirements of each sample. The sequencing library was prepared with a TruSeq Nano DNA LT Library Prep Kit (Illumina). Finally, paired-end sequencing was carried out on a NovaSeq sequencer with a NovaSeq 6000 SP Reagent Kit (500 cycles).

### Bioinformatics analysis

Microbiome bioinformatics were performed with QIIME2 2019.4 with slight modification according to the official tutorials. Briefly, raw sequence data were demultiplexed using the demux plugin followed by primers cutting with cutadapt plugin. Sequences were then quality filtered, denoised, and merged, and chimera removed using the DADA2 plugin. Venn diagram was generated to visualise the shared and unique ASVs among samples or groups using R package “VennDiagram,” based on the occurrence of ASVs across samples/groups regardless of their relative abundance. Alpha-diversity metrics, such as Chao1 richness estimator, observed species, Shannon diversity index, Simpson index, Faith’s PD, Pielou’s evenness, and Good’s coverage were calculated using the ASV table in QIIME2, and visualised as box plots. Beta diversity analysis was performed to investigate the structural variation of microbial communities across samples using Jaccard metrics, Bray–Curtis metrics, and UniFrac distance metrics, and visualised *via* principal coordinate analysis, non-metric multidimensional scaling, and unweighted pair-group method with arithmetic means hierarchical clustering. Microbial functions were predicted by PICRUSt2 upon MetaCyc and KEGG databases.

### Statistical analysis

SPSS 25.0 software was used for statistical analysis. Continuous variables with a normal distribution were expressed as the mean ± standard deviation and were compared *via* the t-test. Continuous variables without a normal distribution were compared *via* the non-parametric test. Categorical variables were described as integers and percentages, and comparisons between groups were performed using Fisher’s exact test. A p-value <0.05 was considered statistically significant.

## Result

A total of 28 infants with heart failure caused by left-to-right shunt congenital heart disease were enrolled as the heart failure group. There were 21 infants with ventricular septal defects, 5 infants with ductus arteriosus defects, and 2 infants with complete endocardial pad defects. The heart failure group comprised 15 male and 13 female infants with a mean age of 2.8 ± 2.9 months, weight of 4.6 ± 1.8 kg, pulmonary artery pressure of 59.8 ± 10.1 mmHg, and NT-proBNP of 4,218.7 ± 3,757.1 pg/ml. Nine of the infants in the heart failure group were exclusively breastfed, 5 were formula fed, and 14 were both breastfed and formula fed. A total of 22 age- and sex-matched children without heart disease served as controls. The control group comprised 12 male and 10 female infants with a mean age of 2.6 ± 1.7 months and weight of 5.1 ± 1.6 kg. Eight of the infants in the control group were exclusively breastfed, 3 were formula fed, and 11 were both breastfed and formula fed. There were no differences in sex, age, weight, or feeding style between the two groups (**Table 1**).

**Table 1:** Comparison of general data between the two groups.

	Intervention group	Control group	P value
Age (month)	2.8±2.9	2.6±1.7	0.745
Weight (kg)	4.6±1.8	5.1±1.6	0.343
Boys/girls	15/13	12/10	0.945
pulmonary artery pressure (mmHg)	59.8±10.1	–	–
NT-proBNP (pg/ml)	4218.7±3757.1	–	–
Feeding methods			
Breastfeeding	9	8	0.904
Formula feeding	14	11	
Mixed Feeding	5	3	

**Figure 1:**
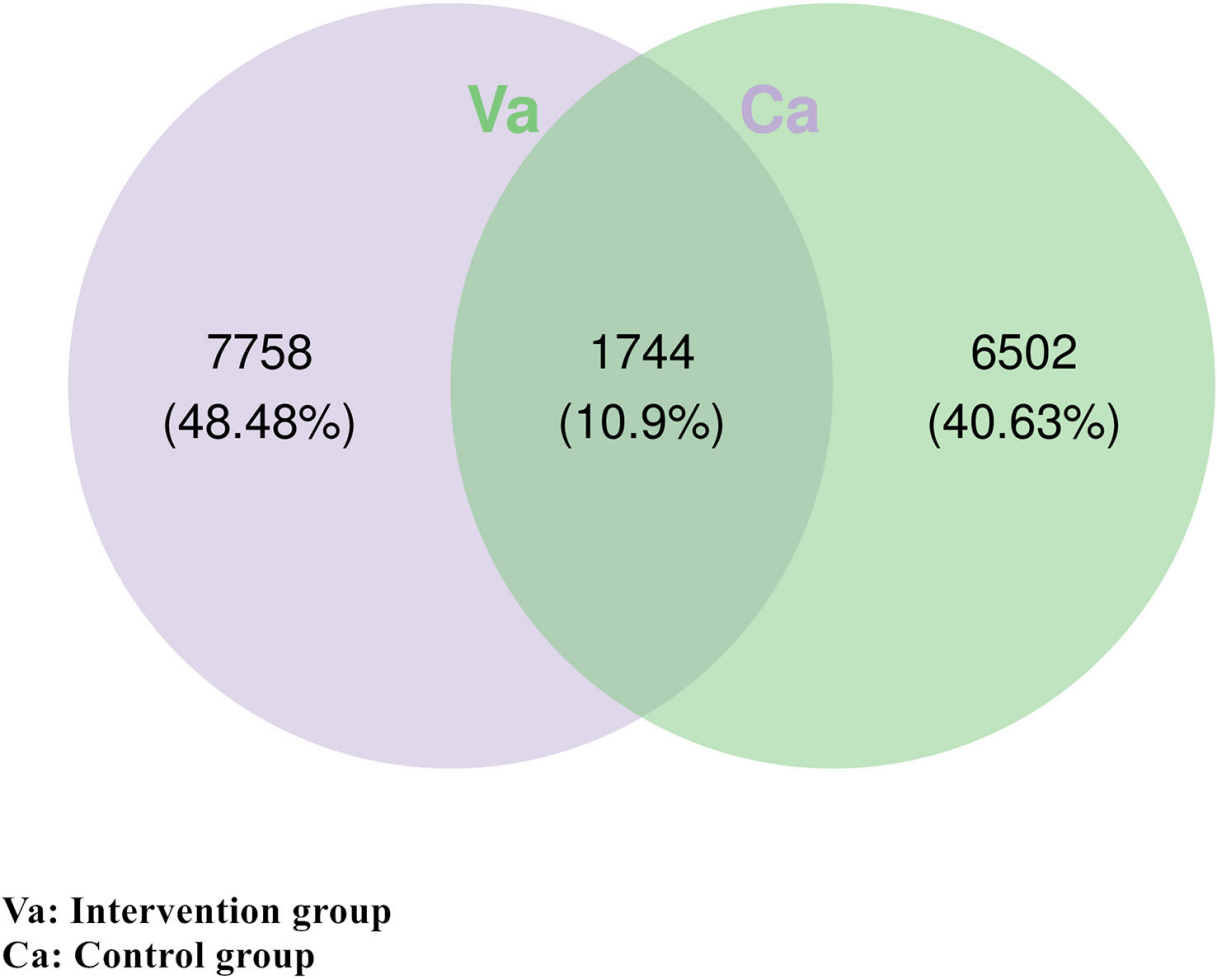
There were 1,744 identical ASVs/OTUs in the heart failure group and the control group. There were 7,758 unique ASVs/OTUs in the heart failure group and 6,502 unique ASVs/OTUs in the control group.

To analyse the differences in intestinal microbial species between the heart failure group and control group, a Venn diagram was constructed using the ASV/OTU abundance table, and the number of members in each set was counted according to presence or absence in the ASV/OTU abundance table. The results showed that there were 1,744 (10.9%) ASVs/OTUs that were shared between the heart failure group and the control group, 7,758 (48.48%) ASVs/OTUs unique to the heart failure group, and 6,502 (40.63%) ASVs/OTUs unique to the control group (**Figure 1**).

**Figure 2:**
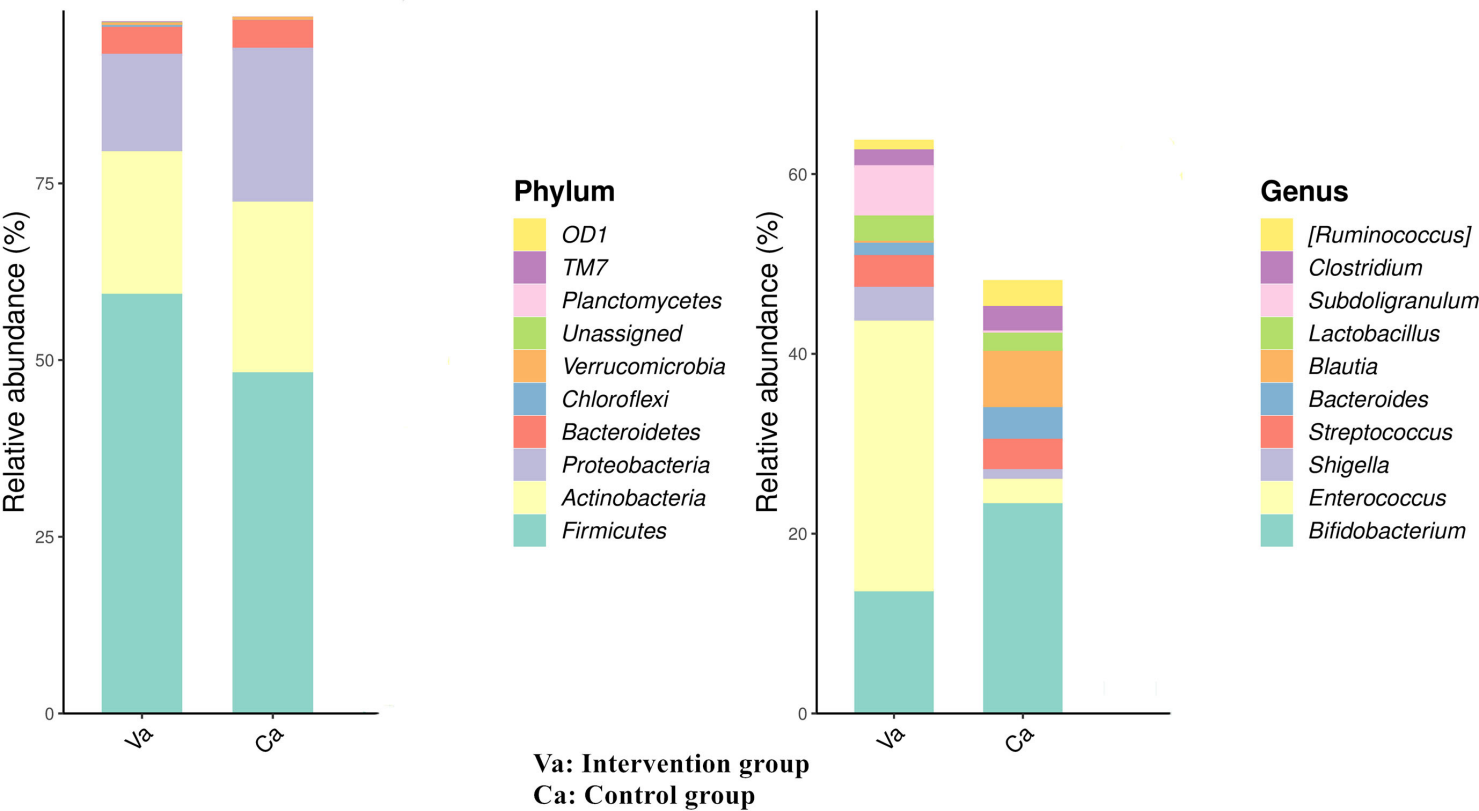
At the phylum level, the most abundant bacteria in the heart failure group were Firmicutes, Actinobacteria, Proteobacteria, and Bacteroidetes, and the most abundant bacteria in the control group were Firmicutes, Proteobacteria, Actinobacteria, and Bacteroidetes. At the genus level, the most abundant bacteria in the heart failure group were *Enterococcus*, *Bifidobacterium*, *Subdoligranulum*, *Shigella*, and *Streptococcus*, and the most abundant bacteria in the control group were *Bifidobacterium*, *Blautia*, *Bacteroides*, *Streptococcus*, and *Ruminococcus*.

We further analyse the species composition and abundance of intestinal microorganisms in the two groups at the phylum and genus levels. At the phylum level, the most abundant bacteria in the heart failure group were Firmicutes (58.8%), Actinobacteria (20.6%), Proteobacteria (10.3%), and Bacteroidetes (3.7%), and the most abundant bacteria in the control group were Firmicutes (47.8%), Proteobacteria (22.1%), Actinobacteria (21.8%), and Bacteroidetes (3.1%). At the genus level, the most abundant bacteria in the heart failure group were *Enterococcus* (30.3%), *Bifidobacterium* (13.1%), *Subdoligranulum* (5.7%), *Shigella* (3.6%), and *Streptococcus* (3.1%), and the most abundant bacteria in the control group were *Bifidobacterium* (24.3%), *Blautia* (6.0%), *Bacteroides* (3.3%), *Streptococcus* (3.0%), and *Ruminococcus* (2.5%) (**Figure 2**).

**Figure 3:**
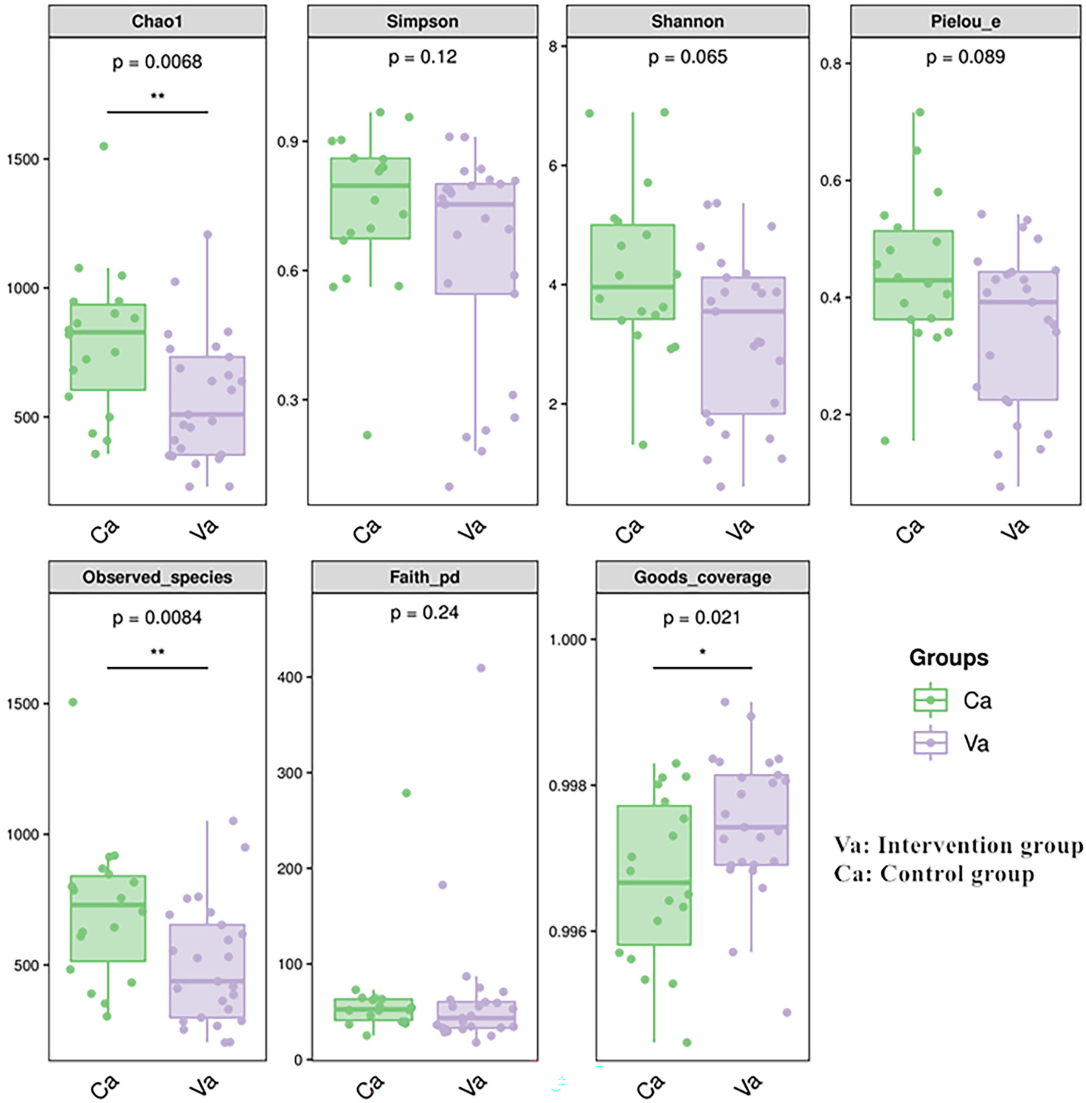
The Chao1 and observed species indices of abundance were significantly lower in the heart failure group than in the control group. The meaning of each symbol in the boxplot was as follows. The upper and lower end line of the box were the upper and lower Interquartile range. Median line of the box was the median. The upper and lower margins were the maximum and minimum internal circumference (1.5 times of interquartile range). Numbers under the diversity index label are p-values.

The comparison of the alpha diversity of the intestinal microbiota between the two groups showed that the Chao1 and observed species indices were significantly lower in the heart failure group than in the control group (**Figure 3**).

**Figure 4:**
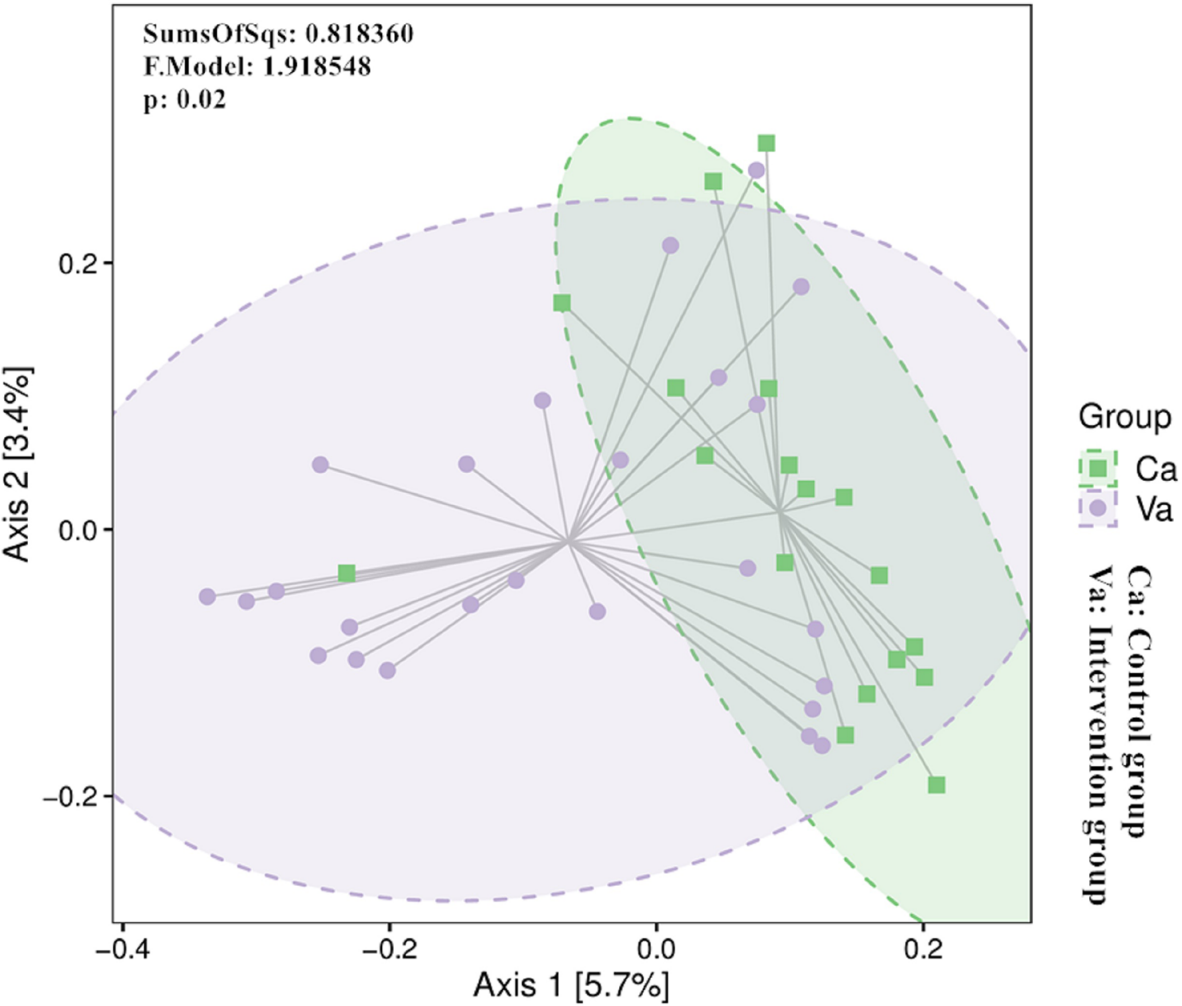
The PCoA of beta diversity based on the Bray−Curtis distance matrix revealed that the differences in community composition between the two groups were significant.

The beta diversity index focuses on the comparison of diversity between different environments, which are represented by infant groups in the present study. We visually analyse the data using principal coordinate analysis, and differences were further examined using Adonis. The PCoA of beta diversity based on the Bray−Curtis distance matrix revealed that the differences in community composition between the two groups were significant (**Figure 4**).

**Figure 5:**
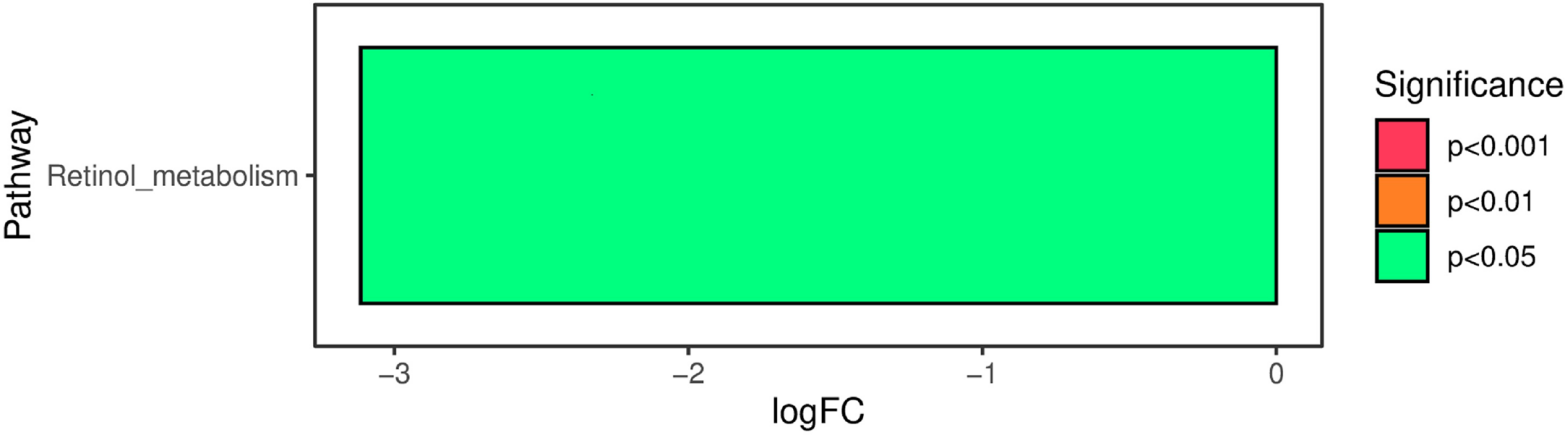
Compared with the control group, retinol metabolism was significantly downregulated in the heart failure group. The negative value of the horizontal axis logFC represents the downregulation of metabolism in the heart failure group compared with the control group. The ordinate represents the different KEGG metabolic pathway labels. The degree of saliency is shown in different colors.

The functional properties of the intestinal microbiota were predicted using PICRUSt2. The analysis of Kyoto Encyclopedia of Genes and Genomes pathway annotations at level 2 as determined by PICRUSt2 revealed significant differences in retinol metabolism between the two groups. Compared with the control group, retinol metabolism was significantly downregulated in the heart failure group (**Figure 5**).

## Discussion

Heart failure is a formidable global public health challenge and is responsible for more than 1 million hospitalisations each year (Jackevicius et al., 2019). Heart failure describes a variety of cardiac structural or functional diseases that result in impaired ventricular filling or ejection capacity, insufficient blood perfusion in organs and tissues, and pulmonary or systemic circulation stasis (Jia et al., 2019). The gut is an endocrine organ that is rich in blood, accounting for approximately 40% of the body’’s total blood, and it is significantly affected by reduced blood supply (Takala, 1996). During the heart failure process, the gut is the first organ to undergo ischaemia and the last organ to recover. Intestinal ischaemia or hyperaemia reduces the intestinal oxygen supply, which can lead to changes in intestinal microbial composition and, in turn, metabolic disorders and intestinal microbiota dysfunction. Furthermore, the intestinal microbiota affects the body’s physiology, including the progression of heart failure (Polsinelli et al., 2019).

The gut contains trillions of microbes—as many cells as the total number of human cells (Sender et al., 2016a). The microbes that colonise the intestinal tract play an important role in the physiological and pathological processes of the body. They participate in nutrient metabolism and absorption, regulate intestinal epithelial barrier function, and affect local or systemic immune inflammatory responses (Nicholson et al., 2012). The composition of the intestinal microbiome is dynamic and may differ in the same individual under different physiological conditions and at different times. These changes may play an important role in human health and disease. Many studies have shown that there is a close relationship between the intestinal microbiota and heart failure (Sandek et al., 2012; Brown and Hazen, 2015; Miele et al., 2015; Marques et al., 2017; Tang et al., 2017; Tang et al., 2019). However, these studies have focused on adults, and few studies have explored the relationship between heart failure and the intestinal microbiota in infants with congenital heart disease. We conducted a cohort study to analyse the relationship between heart failure and intestinal microbiota in infants with congenital heart disease.

Heart failure causes intestinal ischemia or hyperemia, which can result in a reduction in the intestinal oxygen supply. As a result, the intestinal environment changes, which leads to changes in the composition of the intestinal microbiota, mainly a reduction in beneficial intestinal bacteria and an increase in pathogenic bacteria. Chen et al. showed that intestinal microbiota disorder in patients with heart failure manifested as decreases in *Bacteroides* and *Bifidobacteria* and increases in Firmicutes and *Proteus* (Chen et al., 2017). Pasini et al. found increased abundances of intestinal pathogens such as *Candida, Salmonella, Shigella*, and *Campylobacter* in faecal samples from patients with heart failure (Pasini et al., 2016). Sandek et al. also observed excessive growth and adhesion of pathogenic bacteria in the intestinal mucosa of patients with heart failure (Sandek et al., 2007). A similar phenomenon was observed in the present study. Compared with infants without heart disease, the proportion of pathogenic bacteria such as *Enterococcus, Shigella*, and *Subdoligranulum* in the intestinal tract was significantly higher in infants with congenital heart disease with heart failure, while the proportion of beneficial bacteria such as *Bifidobacterium, Blautia*, and *Bacteroides* was significantly lower. Nagatomo et al. and Hooper et al. demonstrated that an increase in pathogenic bacteria and a decrease in beneficial bacteria lead to an increase in inflammatory factors in the intestine and an increase in the body’s inflammatory response, which aggravate the progression of heart failure (Hooper et al., 2012; Nagatomo and Tang, 2015).

The intestinal microbiota coexists with the host. The intestine provides a good colonisation environment for microbes, and the intestinal microbiota plays an important role in maintaining nutrient metabolism, the stability of the intestinal environment, and the health of the body. The intestinal microbiota is the most complex ecosystem in the human body and is composed of a variety of microorganisms residing in the human intestine. These microorganisms coexist mutually in the intestinal tract to jointly maintain the stability of the intestinal environment. Once homeostasis is disrupted, the disorder of the intestinal microbiota will aggravate the pathological state (Sender et al., 2016b). Kummen showed that intestinal hypoxia caused by heart failure reduces the diversity and richness of the intestinal microbiota (Kummen et al., 2018). In severe left-to-right shunt congenital heart disease, heart failure often occurs in infancy due to a large left-to-right shunt, which results in gastrointestinal congestion and disruption of the ecology of the intestinal microbiota. In this study, compared with infants without heart disease, the alpha and beta diversities of the intestinal microbiota were significantly lower in infants with heart failure. These findings indicate that heart failure reduced the diversity of the intestinal microbiota, with a decrease in beneficial bacteria and an increase in pathogenic bacteria, and ultimately aggravated the progression of heart failure.

Many studies have suggested that retinol metabolism is also involved in the progression of heart failure in infants with congenital heart disease. Yang et al. found that cardiac retinoic acid levels decline in heart failure patients (Yang et al., 2021). Osorio found that the protein expression of retinoid X receptor was reduced in pacing-induced heart failure (Osorio et al., 2002). Choudhary showed that retinoic acid can prevent angiotensin II- and mechanical stretch-induced reactive oxygen species generation and cardiomyocyte apoptosis (Choudhary et al., 2008). Subramanian observed that all-trans retinoic acid supplementation can prevent cardiac fibrosis and cytokines induced by methylglyoxal (Subramanian and Nagarajan, 2017). Huang et al. reported that lower levels of serum retinoic acid were associated with more serious cardiovascular disease and higher mortality (Huang et al., 2021). Hence, retinol metabolism was studied in this study. Compared with the control group, retinol metabolism was significantly downregulated in the heart failure group. Disorders of the intestinal microbiota will inevitably lead to changes in the metabolic function of the microbiota, which may cause downregulation of retinol metabolism. Adjusting the intestinal microbiota to upregulate retinol metabolism may be a new way to treat heart failure in infants with congenital heart disease, but further studies are needed.

There are some limitations to this study. First, the main food of infants is milk. In this study, infants were selected as the participants. Although the influence of some foods on the microbiota has been ruled out, interference from differences in formula and the diets of breastfeeding mothers cannot be ruled out. Second, this study only analyse the intestinal microbiota of infants with left-to-right shunt congestive heart failure and did not analyse other types of heart failure. Third, this study was a single-centre study with a small sample size, and a multicentre study with a large sample size should be conducted next.

## Conclusion

There is a close relationship between heart failure and the intestinal microbiota in infants with congenital heart disease. Heart failure in infants with congenital heart disease caused intestinal microbiota disorder, which was characterized by an increase in pathogenic bacteria, a decrease in beneficial bacteria, and decreases in diversity and richness. We also found that retinol metabolism of the intestinal microbiota was significantly downregulated in infants with heart failure, which may be related to the progression of heart failure. The underlying mechanism needs to be studied further.

## Data availability statement

The datasets presented in this study are deposited in online repositories. The names of the repositories and accession numbers can be found below: https://www.ncbi.nlm.nih.gov/bioproject/?term=PRJNA862324.

## Ethics statement

The studies involving human participants were reviewed and approved by Fujian Children’s Hospital. Written informed consent to participate in this study was provided by the participants’ legal guardian/next of kin.

## Author contributions

Q-LZ designed the study, acquired and interpreted the data, and drafted the manuscript. X-HC analysed and interpreted the data. S-JZ,acquired and analysed the data. J-SH and Y-QL collected and analyse faeces specimens. QC has made substantial contributions to conception and design. HC was involved in analysis and interpretation of data, revision of the manuscript, and given final approval of the version to be published.
